# New RNA-Based Breakthroughs in Alzheimer’s Disease Diagnosis and Therapeutics

**DOI:** 10.3390/pharmaceutics13091397

**Published:** 2021-09-03

**Authors:** Micaela Riscado, Bruno Baptista, Fani Sousa

**Affiliations:** CICS-UBI—Health Sciences Research Centre, University of Beira Interior, Av. Infante D. Henrique, 6200-506 Covilhã, Portugal; micaela.riscado@ubi.pt (M.R.); bruno.baptista@ubi.pt (B.B.)

**Keywords:** Alzheimer’s disease, amyloid hypothesis, dementia, diagnosis, RNA-based therapeutics

## Abstract

Dementia is described as the fifth leading cause of death worldwide and Alzheimer’s disease (AD) is recognized as the most common, causing a huge impact on health costs and quality of patients’ lives. The main hallmarks that are commonly associated with the pathologic process are amyloid deposition, pathologic Tau phosphorylation and neurodegeneration. It is still unclear how these events are linked to the disease progression, due to the complex pathologic mechanisms. Nevertheless, several hypotheses have been proposed for a better understanding of AD. The AD diagnosis is performed by using a combination of several tools to detect β-amyloid peptide (Aβ) deposits and modifications in cognitive performance, sometimes being expensive and invasive. In the treatment field, there is still an absence of effective treatments to delay or stop the progression of the disease, with most of the approved drugs used to relieve symptoms, and all of them with significant adverse side effects. Considering all limitations, the need to establish new and more effective diagnostic and therapeutic strategies becomes clear. This review aims not only to describe the disease and its impact but also to collect the currently available diagnostic and therapeutic strategies, highlighting new promising RNA-based strategies for AD.

## 1. Dementia and Alzheimer’s Disease Impact

**Figure 1:**
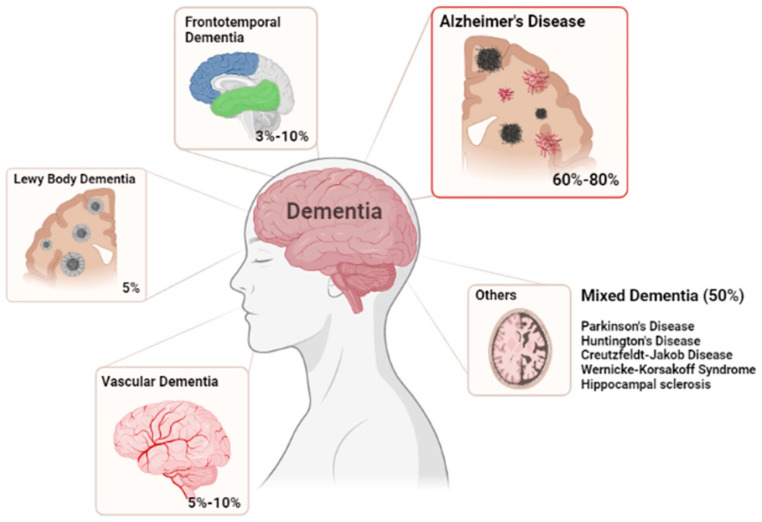
Most common forms of Dementia. Illustrating the correspondent percentages of cases for each disease.

According to The World Health Organization, presently, dementia is the 7th largest cause of death in the world [1]. Dementia is a generic term used to designate a wide range of diseases, with Alzheimer’s disease (AD), Vascular Dementia, dementia with Lewy bodies, Frontotemporal Dementia and Parkinson’s Disease being the most relevant [2,3,4] (Figure 1). Dementia is usually associated with a group of symptoms affecting memory, thinking and social abilities, severe enough to interfere with daily life [5]. In general, the most characteristic symptoms, rarely treated or prevented efficiently are the deterioration of cognitive performance; behavioral disturbances; intellectual loss; difficulty in solving problems; loss of other cognitive skills affecting daily activities; and ultimately death [2,5,6,7,8]. Age is indicated as the biggest risk factor for the development of dementia. It is estimated that dementia affects approximately 47 million people worldwide and that by 2050, this number can reach about 131 [9]. The increased prevalence of these diseases is mainly associated with population aging, due to increased life expectancy. The number of people affected and the high impact that these diseases have on the quality of life are important arguments to make dementia treatment an attractive opportunity for pharmaceutical companies [5,10]. The World Health Organization has recognized AD as the most common and devastating form of dementia of our time, where two-thirds (50–75%) of the people affected can face death in approximately 8.5 years after the onset of symptoms [5,10]. AD is a multifactorial, progressive, chronic neurodegenerative disorder that occupies the 3rd place in the diseases that causes disability and death for the elderly, after cardiovascular/cerebrovascular diseases and malignant tumors [5,9,11]. It is estimated that about 31 million people have AD worldwide, and due to advances in healthcare, life expectancy has increased, which can contribute to the expected increase in AD cases in the next few years. Importantly, AD is also a comorbidity to other severe human diseases and is associated with high healthcare costs—it is estimated that the overall cost of health and social care could reach 2 trillion dollars by 2030 [9,10,12,13].

## 2. Alzheimer’s Disease Characterization

Alzheimer’s disease was first described by Dr. Alois Alzheimer in the early 20th century. The patient, “Auguste D.”, experienced memory loss, paranoia, and psychological changes. In postmortem evaluation, shrinkage in and around nerve cells was detected in the patient’s brain [14]. The pathological hallmarks of AD were first described in 1906 as being extracellular plaques, intercellular tangles, and widespread neurodegeneration in the brain. Decades later, the β-amyloid peptide (Aβ) and Tau were identified as the main constituents of these tangles and plaques [15].

Generally, the risk factors for AD can be divided into two types: modifiable and non-modifiable. The modifiable factors include poorly controlled type 2 diabetes, cardiovascular diseases (like stroke, hypertension), depression, traumatic brain injury, lifestyle, and environmental factors (including stress, alcohol consumption, smoking, high blood pressure, high cholesterol, obesity, and lack of exercise). In turn, the non-modifiable factors include genetic mutations, genetic polymorphisms, age or gender [10,12,16]. 

**Figure 2:**
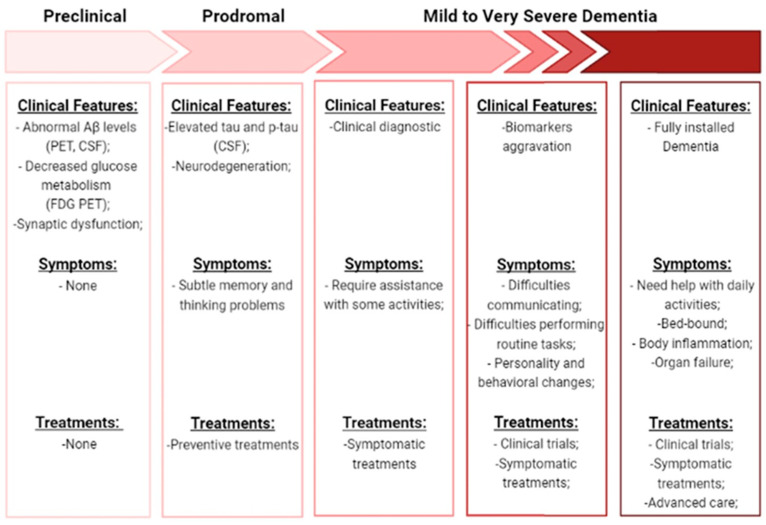
Description of the progression along the seven stages of AD. Is shown the clinical features, symptoms, and treatments specific to each stage. (Aβ—amyloid beta-peptide; PET—positron emission tomography; FDG—18F-fluorodeoxyglucose; CFS—Cerebrospinal fluid; p-Tau—phosphorylated-Tau).

These risk factors can lead to a progression along the seven stages associated with Alzheimer’s disease: preclinical (positive biomarkers but no cognitive impairment), prodromal (very mild cognitive impairment), mild dementia, moderate dementia, moderately severe dementia, severe dementia, and very severe dementia [4,17]. These stages and their features are summarized in Figure 2. Studies of biomarkers and PET scans suggest that signs associated with AD may be found in the patient’s brain 20 years before the first symptoms appear. When some changes occur and are no longer reversible, symptoms gradually appear [10,16]. Behavioral changes, impaired mobility, hallucinations, and seizures are the first cognitive decline. Then, memory loss occurs, and in more serious cases, basic daily functions start to be affected, leading to the inability of independently living. In the end, other clinical syndromes also emerge like posterior cortical atrophy (PCA), logopenic aphasia (LPA), and AD frontal variant, leading to death [5,12,18]. Histopathological and morphological examination of AD postmortem brains in combination with studies on AD transgenic mouse models show multiple cellular changes. Cerebral amyloid angiopathy, neurofibrillary tangle, senile plaques, inflammation (microglial activation) and oxidative stress are the most prominent features identified. Cholinergic neuron damage, dystrophic neurites, astrogliosis and altered acetylcholine levels are other cellular changes well established in AD. These main changes can consequently result in mitochondrial fragmentation, mitochondrial DNA damage, and hormonal imbalance. Downstream consequences of these processes include neurodegeneration with synaptic and neuronal loss, leading to macroscopic atrophy. These alterations are primarily observed in the learning and memory regions of the brain, including the entorhinal cortex and spread regions of the hippocampus, temporal cortex, frontoparietal cortex and subcortical nuclei [5,10,12,18]. Therefore, the three biomarkers that are commonly used to document the underlying pathologic processes of AD are mainly: amyloid deposition, pathologic Tau (microtubule-associated protein) and neurodegeneration. Although the clinical characteristics and severity are better correlated with neurofibrillary tangles (NFT), data suggest that Aβ pathology develops many years before clinical symptoms appear and precedes Tau changes [19,20]. Once the presence of the pathology of Tau can be related to the normal healthy aging process. It is still unclear how Aβ and Tau are mechanistically linked, but some studies suggested that this interaction occurs in the immune system, since activated microglia co-localize with amyloid plaques and some AD-risk genes are involved in immune system pathways [5,21]. It is thought that Aβ deposition leads to microglia activation and astrocytes reactivation in AD, causing pro-inflammatory cytokines release (TNF-a or IL-6), which in turn can lead to Tau hyperphosphorylation and neuronal damage [22]. Due to the complexity of this disease, the study of multiple molecular targets, mechanisms, and pathways is still necessary, but several hypotheses have been proposed for a better understanding of AD [10,16,23].

### 2.1. Sporadic AD vs. Familiar AD

AD is commonly classified into two types, sporadic AD that can appear at any time in life but usually appearing after 65 years old, and familial AD that appears early-onset, between 30 and 50 years old. Sporadic AD is the most abundant and poorly understood AD form, but it is thought that it may result from a combination of genetic (70%) (some examples are mentioned in Section 3.2), and environmental factors (30%) (inflammation, cholesterol metabolism and endosomal vesicle recycling pathways) [5,18,24]. The extremely uncommon form, representing 1 to 5% of AD cases, is the inherited autosomal, dominant (familial) AD, with clinical symptoms similar to sporadic AD, namely disease progression, and biochemical and neuropathological changes (abnormal overproduction of Aβ) [10,23]. This form is caused by mutations in three genes coding for amyloid precursor protein (APP), presenilin1 (PSEN1), and presenilin 2 (PSEN2) proteins, which are linked to Aβ processing by γ-secretase complexes [5,10,12].

### 2.2. Amyloid and Tau Hypotheses

The most popular theories for explaining the AD pathological pathway are the amyloid and Tau hypotheses. The hypothesis of the amyloid cascade was proposed by Hardy in the early 1990s, where it was described that Aβ peptides sequentially led to the formation of neurotoxic oligomers, insoluble amyloid fibrils, and finally, amyloid plaques [25]. The amyloid peptide is generated by the cleavage of the transmembrane APP. This protein, APP, has non-pathogenic functions and plays vital physiological roles in metal metabolism, by having metal-associated redox activity and by stabilizing the plasma membrane for iron transport, also impacting the modulation of synaptic functions and neuronal survival. In the amyloidogenic pathway, APP is cleaved by the β-secretase (BACE), an aspartyl protease with two isoforms BACE1 and BACE2, leading to the formation of sAPPβ, that is further cleaved by γ-secretase complex to produce Aβ. The γ-secretase complex is composed of four subunits, including presenilin (PS) 1 and 2, nicastrin, anterior pharynx defective 1, and presenilin enhancer 2. Presenilin comprises the catalytic domain of γ-secretase, and the PS1 dysfunction has been directly linked to AD [21,26]. γ-secretase cleavage is inconsistent, resulting in differences at the C-terminal end of the generated peptides. These differences contribute to the existence of a variety of Aβ isoforms. The Aβ1-40 and Aβ1-42 are the most common isoforms, corresponding to the cleavage at the 40 and 42 positions, respectively. Moreover, Aβ1-40 is the most abundant form, while Aβ1-42 is slightly longer and less abundant, but is more hydrophobic and fibrillogenic, making it the main species accumulated in the brain of AD patients [10,12,21,26].

So far, it is well known that almost all APP cluster mutations occur around the β-secretase and γ-secretase cleavage sites [27]. 

**Figure 3:**
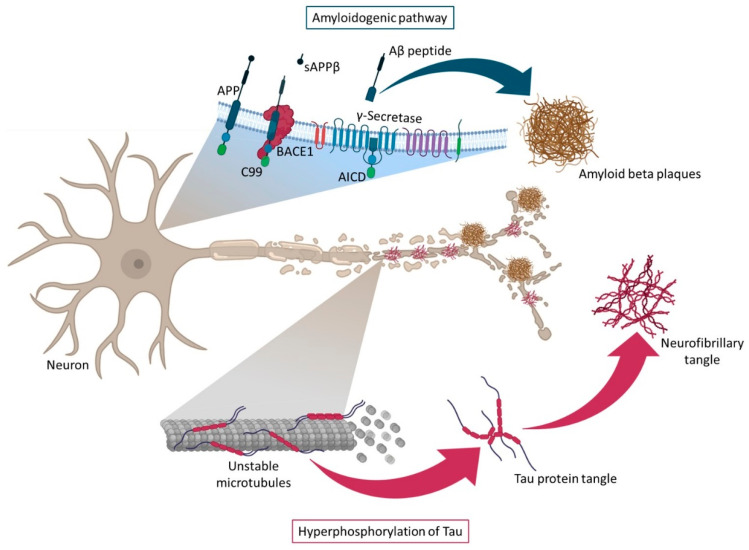
Representation of the amyloidogenic pathway and Tau protein hyperphosphorylation. The formation of β-amyloid plaques (amyloidogenic pathway) occurs in the cell membrane and is due to the cleavage of APP by β-secretase, which gives rise to two products: a soluble APP fragment (sAPPβ), which is released into the extracellular space and a membrane-anchored C-terminal fragment (C99). This is later cleaved by γ-secretase, originating APP intracellular domain (AICD) and Aβ peptides, that further forms oligomers and, eventually, accumulate in Aβ plaques. The formation of neurofibrillary tangles (hyperphosphorylation of Tau protein) occurs due to hyperphosphorylation of Tau protein, which loses affinity for microtubules and thus causes their disintegration in AD.

Although the exact pathogenic role of Aβ is unknown, it is well documented that Aβ toxicity depends on size, state of aggregation, and diffusion in subcellular compartments and neuronal terminals. The pathogenicity of Aβ is amplified when monomers become oligomers, leading to plaques formation. The intraneuronal accumulation of Aβ peptides and amyloid plaques lead to a large number of neurotoxic processes such as loss of mitochondrial function, generation of reactive oxygen species (ROS) increasing oxidative stress, disruption of calcium homeostasis, activation of microglia causing neuroinflammation and neuritic alterations/synaptic distortions in cortical regions closer to the Aβ plaques. Furthermore, these neurotoxic events act as positive feedback, making it impossible to restore the original balance. In turn, there is evidence suggesting that Aβ plays a role in inducing Tau hyperphosphorylation, leading to the formation of protein tangles, the second hallmark feature of AD [12,16,21]. Tau protein is a highly soluble microtubule-associated protein (MAP) encoded by the MAPT gene.One of the Tau main functions is to modulate the stability of axonal microtubules, once the microtubules are naturally unstable and require interaction with Tau to maintain their structure. Tau hyperphosphorylation results in disruption of microtubule organization [16,28]. In a normal brain, there is a balance between phosphorylation and dephosphorylation of the Tau protein, which allows maintaining the stability of the cytoskeleton and, consequently, the axonal morphology of neurons. However, under pathological conditions, the accumulation of hyperphosphorylated Tau in neurons leads to protein misfold and aggregation in intracellular NFTs, reducing their affinity for microtubules [10,16,18,21,29]. The loss of normal Tau protein function leads to a pathological disturbance in the structural and regulatory functions of the cytoskeleton of neuronal cells, causing alterations in the morphology, neuronal plasticity, axonal transport, and provoking synaptic dysfunction and neurodegeneration [30]. This hyperphosphorylation of Tau is especially caused by the increased activity of kinases, namely glycogen synthase kinase-3 (GSK-3), CDK5, and the MAP/microtubule affinity-regulating kinase (MARK) which are activated by Aβ oligomers [2,28,30,31,32]. However, the inhibition of some phosphatases, including protein phosphatase 2A (PP2A) and calcineurin, also plays a crucial role, culminating in the formation of NFTs [28,30,33,34,35]. In general, significant evidence supports an Aβ-centered view of AD, and more research is still needed to understand if these two hypotheses are independent or inter-related paths. Figure 3 represents the main events associated with these two ideas. However, more recently, an additional perspective has been included in the AD-research equation with an increased interest linked to the study of neuroinflammation in AD.

### 2.3. Neuroinflammation, Oxidative Stress, and Autophagy in AD

**Figure 4:**
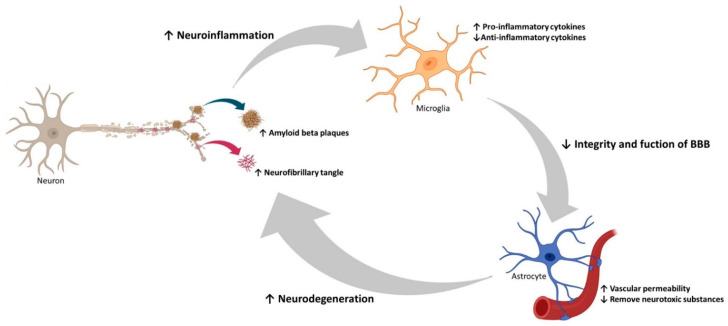
Overview of neuroinflammation, oxidative stress, and autophagy in AD. Representation of the consequences of Aβ plaque formation, namely, hyperphosphorylation of Tau protein, increased oxidative stress, inflammatory responses by microglia activation, damage to astrocytes, which lead to BBB disruption, causing cerebrovascular damage, dysfunction, and decreased autophagy.

Emerging studies have been focusing on other pathological paths that may have an important role in AD. Neuroinflammation, oxidative stress, and autophagy dysregulation have been proven to be present and to have an important role in the course of the disease (Figure 4) [36]. The presence of Aβ and the occurrence of mutations in genes encoding for the innate immune system molecules provokes microglia activation. These cells become more susceptible to stimulus and produce, continuously, inflammatory cytokines and chemokines that lead to pro-inflammatory, cytotoxic events, and Tau protein hyperphosphorylation. All this contributes to the disruption and deterioration of the blood–brain barrier (BBB) [37,38,39], which causes increased vascular permeability and inability to remove neurotoxic substances from the CNS, such as Aβ peptides and hyperphosphorylated Tau. Consequently, neuroinflammatory responses will happen, which further contribute to neurodegeneration, taking place in a feed-forward loop [37,40,41]. Additionally, in the aging process, some endogenous and external environmental stimuli, increase free radicals, mainly reactive oxygen and nitrogen species, triggering an imbalance of the oxidation-antioxidant system. This imbalance affects cells either by cellular dead or dysfunction [42,43]. Additionally, autophagy, a process responsible for the clearance of abnormal proteins and components of cells, is thought to play an important role in AD, when it is dysregulated. Accumulation of autophagosomes, Aβ, and phosphor-Tau are neuropathological features of AD, that can come from the dysregulation of this process [44]. Nassif and Hetz demonstrated that in autophagy-deficient mice, phosphor-Tau was accumulated, and they suggested that restoring autophagy could reduce this abnormal accumulation [45]. Nevertheless, these three mechanisms are far from being well-established and more in-depth investigation is required to clarify this complex disease.

## 3. Diagnostic Tools for Alzheimer’s Disease

### 3.1. Approved Diagnostic Tools

A simple view of the complex pathologic pathway of AD can be described by considering that the disease is initiated by Aβ plaques deposition, followed by accumulation of Tau tangles and eventually by neurodegeneration. If the cascade of events follows or not exactly these steps, is still a subject under study. Nevertheless, this simple view of events is the basis of current AD diagnostic tools, focused on the characterization of proteins that have a role in the pathophysiology of AD at a specific stage and on the evaluation of neurodegeneration markers. So, for AD diagnosis, a combination of several tools is used, which include the clinical examination by magnetic resonance imaging (MRI) for the mesial temporal lobe atrophy or, more recently, functional-connectivity MRI; positron emission tomography (PET) to detect Aβ deposits, Tau presence or abnormal brain metabolism by 18F-fluorodeoxyglucose (FDG); Cerebrospinal fluid (CSF) assays to detect Aβ42, total Tau, threonine 181 (T181) phosphor-Tau and neurofilament light chain; and neuropsychological tests to assess cognitive performance [10,18]. These diagnostic markers allow the recognition of AD patients and their categorization at different stages. Decreased CSF levels of Aβ42 and increased amyloid PET signal come before subtle cognitive impairment (neurodegeneration and synaptic dysfunction). MRI detection of hippocampal volume loss and high concentrations of total Tau and/or phospho-Tau, in CSF, can predict the beginning of AD pathology and clinical presentation [46,47,48]. Nevertheless, these techniques are invasive and expensive making it difficult to apply them in routine clinical practice. Thus, despite all progress on diagnostic processes, postmortem gross specimen analysis and histology of brain sections, for amyloid plaques and neurofibrillary tangles evaluation, continues to represent a high percentage of AD neuropathologic analysis [18,46]. The absence of effective treatment for AD makes even more important the early diagnosis. Thus, developing new, accessible, specific, and sensitive molecular biomarkers and diagnosis methods is mandatory to anticipate the disease, with great impact on the health economy and quality of life.

### 3.2. Novel Diagnostic Approaches

**Figure 5:**
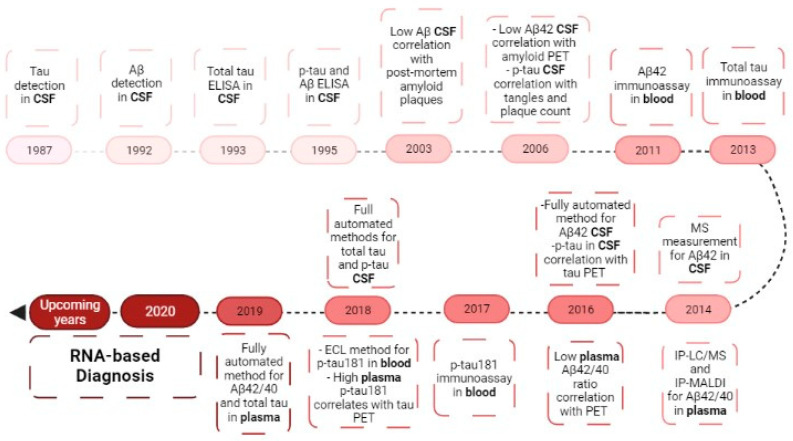
Biomarkers evolution in the diagnosis of Alzheimer’s disease. Evolution from the study of CSF biomarkers to the blood biomarkers, ending in the RNA research of today. CSF—cerebrospinal fluid; Aβ—amyloid beta-peptide; p-Tau—phosphor-Tau; PET—positron emission tomography; MS—mass spectrometry; IP-LC—immunoprecipitation liquid chromatography-mass spectrometry; IP-MALDI—immunoprecipitation matrix-assisted laser desorption/ionization; ECL—electrochemiluminescence.

New tools for AD diagnosis are expected not only to allow easy detection of preclinical stages of AD but also permit monitoring disease progression and treatment response. Tissue biopsies are too invasive, so the new diagnostic studies focus on genetic, circulating, and imaging-based biomarkers [46]. Genetics is still a poorly understood territory that represents small contributions to the overall disease. Genes more likely to undergo genetic alterations that are correlated with AD may be sortilin-related receptor 1, clusterin, complement receptor 1, CD2-associated protein, ephrin type-A receptor 1 and membrane-spanning 4-domains subfamily A, among others [12,24]. In familiar AD, APP, PSEN1, and PSEN2 mutations account for approximately 30–50% of the cases and in sporadic AD, the apolipoprotein ε4 allele (APOE4) increases the risk by 20–30% [46]. APOE isoforms have been difficult to determine, but it is known that they can promote Aβ aggregation and impair Aβ clearance in the brain. Moreover, APOE can also participate in the regulation of glucose metabolism, neuronal signaling, and Tau-mediated neurodegeneration [10,12,16]. In, 2017, the 23andme company received FDA approval to diagnose the ApoE allele and communicate with patients the increased probability to develop AD [49]. More recently, the identification of a novel APP gene allele, lead to the discovery of the Alzheimer Associated protein (ALZAS), which is overexpressed in the blood of patients affected by AD and can become a novel biomarker [49]. Imaging-based biomarkers had a big breakthrough with the amyloid and Tau PET imaging, but the sensitivity of the amyloid PET ligands remains to be determined and further studies are necessary to correlate Aβ and Tau PET imaging results to CSF biomarkers and cognitive measures. Nevertheless, the FDA recently approved a dopamine transporter (DAT) single-photon emission computerized tomography (SPECT) to evaluate suspicions of Parkinson’s disease, which can be seen as evidence of the next imaging-based diagnostic in AD [46]. These two groups of biomarkers can be less invasive but continue to be expensive and hard to include in routine clinical practice. So, to try a more accessible approach, recent studies focus on circulating biomarkers as the next promising tool in the diagnostic area [50,51]. The easier way to assess patients’ samples for biomarkers study is through the blood. CSF is the biofluid closest to the CNS cells; however, to be obtained, an invasive lumbar puncture is needed [46]. On the other hand, blood is a rich source of molecules, including RNA, originating from different tissues in the human body. Figure 5 shows the biomarkers evolution in AD diagnostic. Certain circulating levels of these molecules are altered in some pathological conditions, allowing one to draw some conclusions about the processes that are happening in the cells [46,47,52]. Before the passage through BBB, proteins/peptides suffer cleavage, and metabolites pass passively or through portal systems at differential rates. Additionally, in the blood molecules suffer metabolization into different products. So is necessary to keep in mind that some molecules can be intact, and others can become different from the form present in the brain. Recent biomarker development efforts for AD have focused on the characterization of circulating RNA that can influence the regulation of genes involved in AD [46,47,49,52]. 

#### 3.2.1. Ribonucleic Acid

**Figure 6:**
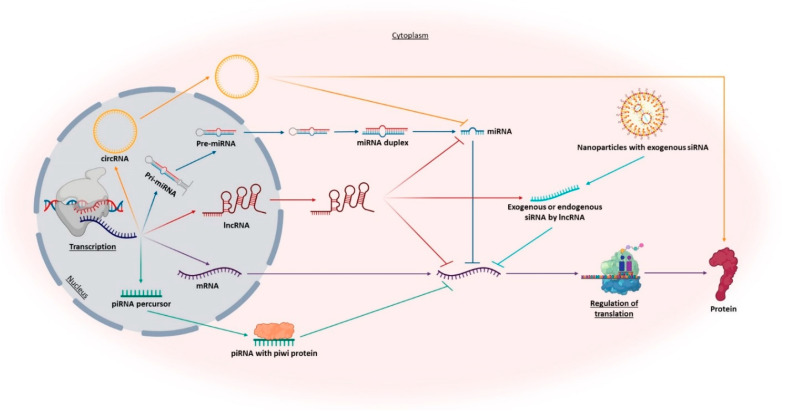
Representation of the roles of different promising RNAs in AD diagnosis and therapy. Including messenger RNA (mRNA), long ncRNAs (lncRNAs), circular RNAs (circRNAs), microRNAs (miRNAs), piwi-associated RNAs (piRNAs), and endogenous and exogenous small interfering RNAs (siRNAs).

Ribonucleic acid (RNA) is a polyanionic macromolecule, with a single chain of four different nucleotides, make it a simple source of genetic information, comparing to the double chain of the DNA [53]. In 1990, Andrew Fire and Craig Mello discovered the RNA interference (RNAi) [54] and showed that the RNA is more than a simple intermediate molecule in the genetic information transfer from DNA to proteins, allowing the world to show interest in other types of RNA besides the well know messenger RNA (mRNA), transfer RNA (tRNA) and ribosomal RNA (rRNA). RNA is currently recognized as a fundamental molecule for the regulation of gene expression [53]. This regulation occurs at a post-transcriptional level, through non-coding RNA molecules (ncRNAs) by blocking the translation or inducing the degradation of their target mRNA via sequence-specificity [55]. There is a large group of ncRNAs-regulated gene sequences, some of which playing important roles in a variety of diseases. For example, in neurodegenerative disorders, dysregulated levels of ncRNAs can be a consequence of some imbalance in their expression and will also result in target proteins dysregulation [21,56]. ncRNAs can be classified into two main groups according to their length (Figure 6). If they have above 200 nt are called long non-coding RNAs (lncRNAs) and when they present circular form, are called circular RNAs (circRNAs). If they have less than 200 nt are named small non-coding RNAs (sncRNAs). Both types of ncRNAs are particularly abundant in the central nervous system [21,57,58]. For classification and characterization, sncRNAs can be subdivided via biogenesis and mode of action into infrastructural RNAs [rRNA, tRNA, small nuclear RNA (snRNA), small nucleolar RNA (snoRNA)] and regulatory RNAs [microRNA (miRNA), small interfering RNA (siRNA), short hairpin RNA (shRNA) and PiWI-interacting RNAs (piRNAs)] [58,59]. Figure 6 summarizes some RNA functions, more related to AD diagnosis and therapeutics.

#### 3.2.2. Novel RNA-Based Diagnostic Tools

Extracellular environments harbor a vast range of RNAs, that remain stable against blood RNases. It is not yet clear how circulating RNAs stay stable but is speculated that extracellular vesicle-like exosomes might play an important role in that stabilization [48,52]. MicroRNAs are the most studied species for AD diagnosis due to their unique characteristics, such as the possibility to correlate the miRNAs levels with their activity (as they are not translated) and their presence in peripheral biofluids. Recent data suggest that circulating miRNAs are representative of releasing tissues, allowing one to understand what is happening inside the cells [6,60,61,62]. In the case of AD, several studies suggest that specific miRNAs can play an important role in pathogenesis and appear to be dysregulated in the blood of AD patients. Additionally, Leidinger and collaborators defined circulating miRNA profiles, specific for AD, discriminating AD from controls with 93% of accuracy, and from other neurological diseases like schizophrenia, depression, and bipolar disorder with about 76% of accuracy, as summarized in Table 1 [63].

A different study showed that in extracellular vesicles of serum of AD murine, miRNA-193b, an APP expression negative regulator, was significantly decreased when compared to the wild-type mice [64]. Cheng and collaborators also identified a panel of 15 differentially expressed miRNAs in serum extracellular vesicles that correlate with APOEε4 status. This allowed predicting AD with a sensitivity of 87% [65]. These studies prove that miRNA can be the next generation of AD biomarkers. Indeed several clinical trials currently ongoing have miRNAs as target biomarkers [66], as summarized in Table 2.

In this field of RNA-based diagnosis, some studies focused on lncRNA dysregulation and its role in certain diseases, such as AD and several types of cancer. In the cancer biomarker research area, lncRNAs have been vastly investigated [67]. For example, the lncRNA PCA3 is elevated in patients with prostate cancer and due to its stability in biological fluids, PCA3 can be easily detected in urine. This is a non-invasive biomarker specific to prostate cancer that was approved for clinical trials [68]. In AD, lncRNA 51A and BACE1-AS overexpression has been detected in patients, which can make them potential diagnostic biomarkers for this disease [69,70]. Fotuhi and collaborators showed that lncRNA BACE1-AS is upregulated in the plasma of AD patients and can show some differences between pre-AD and full-AD patients [71]. Modarresi group, in 2011, studied BACE1 and BACE1-AS levels in AD models of young mice and aged mace. In the early stage of AD, the young mouse showed lower BACE1 and BACE1-AS levels, consequently having less Aβ aggregation. In the aged AD mouse, BACE1 and BACE1-AS showed an elevated expression with increased levels of insoluble aggregated Aβ oligomers [72]. This study suggested that BACE1 and BACE1-AS levels can be correlated. Additionally, circRNAs have shown potential to be used as biomarkers. circRNAs are evolutionarily conserved, endogenous non-coding circular RNAs, abundantly expressed in eukaryotes. Like the other ncRNAs mention before, they are stable, but due to their closed circular conformation, they can be even more stable in blood [58,73,74]. Dube and colleagues have shown that the expression of circRNAs changes before the appearance of significant onset symptoms of AD, demonstrating that circRNAs levels can be correlated with neuropathological and clinical evaluation of AD severity [75]. An example includes the circRNA KIAA1586, which is significantly upregulated in AD-associated biological processes and may be a novel risk factor in the pathogenesis of AD [76]. Moreover, circ-AXL, circ-GPHN, and circ-PCCA differ significantly between AD patients and normal controls when studying the expression profile in cerebrospinal fluid, which shows their potential as biomarkers in AD [77]. Overall, the dysregulated and complex ncRNAs levels are closely associated with core pathophysiological processes of AD via regulating gene expression. Because ncRNAs are widely expressed in the brain and show a range of differences between AD and healthy controls, it can be hypothesized that these RNAs are potentially the next generation of AD diagnostic tools.

## 4. Therapeutic Applications for Alzheimer’s Disease

### 4.1. Approved Therapeutics

Treatments for AD have two main goals: (1) relieving cognitive symptoms, to improve or maintain cognitive and daily activity skills; and (2) slowing the progress of the disease. Until this year only a few drugs were approved by FDA and all of them were directed to stabilize symptoms for a limited time [12,16,18,78]. In 1970, the susceptibility of the cholinergic system was identified, leading to the emergence of the first effective drug for the treatment of cognitive symptoms of AD, tacrine (Cognex^®^, Parke-Davis, Detroit, MI, United States). However, tacrine was withdrawn from the market due to its side effects in the cholinergic system and liver toxicity, but it paved the way to other cholinesterase inhibitors being exploited [79,80,81,82]. Cholinesterase inhibitors are used in patients with mild to moderate AD, improving neurotransmission by acetylcholinesterase inhibition (hydrolysis of acetylcholine) in the synaptic cleft, consequently increasing the levels of acetylcholine. Donepezil (Aricept^®^, Eisai and Pfizer, Woodcliff Lake, NJ, United States), Rivastigmine (Exelon^®^, Novartis, Basel, Switzerland), Galantamine (Razadyne^®^, Janssen, Beerse, Belgium) are currently approved drugs, with small but valuable clinical benefits. However, they can also cause adverse effects such as nausea, diarrhea, and vomiting [10,83,84,85,86]. Another drug available to treat the cognitive problems of AD is a glutamate regulator, Memantine (Namenda^®^, Allergan, Dublin, Ireland). This N-methyl-D-aspartate (NMDA) receptor antagonist is prescribed for moderate-to-severe AD. The NMDA receptor is abundant in areas involved in cognition, learning, and memory. Memantine, the NMDA antagonist, has a moderate affinity that allows the physiological action of glutamate (NMDA ligand) without receptors overactivation. In some cases, the combination of memantine and donepezil (Namzaric^®^, Allergan, Dublin, Ireland) [10,83,86,87,88] is also used. This year, 2021, FDA approved a new drug, aducanumab (Aduhelm™, Biogen, Cambridge, MA, United States), which is the first drug to address the underlying biology of AD. This drug is a human IgG1 monoclonal antibody that binds to Aβ fibrils and soluble oligomers, leading to a dose-dependent reduction in Aβ and some reduction in CSF phosphorylated-Tau [89]. In addition, one of the drug classes prescribed for AD patients is antipsychotics due to changes in their behavior, however, haloperidol, suvorexant (Belsomra^®^, Merck, Darmstadt, Germany), and other antipsychotics have severe side effects, like sedation, leading to physical injuries [83]. An alternative treatment is the use of antioxidants like selegiline, alpha-tocopherol (vitamin E), and vitamin D, even not showing consistent benefits for patients. On the contrary, nutraceutical huperzine A seems to show benefits in memory and daily activities [83,86]. Furthermore, there are also non-drug treatments that may be recommended for AD patients, like the Mediterranean diet, regular aerobic exercise, and recreational physical activity [83,86]. Cognitive training or stimulation also show improvement in cases of depression, anxiety, and aggression, improving the quality of life. In general, non-drug treatments show some effect in behavioral symptoms, avoiding that way the use of antipsychotics [90]. Considering all these reasons, limitations, and adverse effects, it becomes clear the need to establish new therapeutic strategies, focusing on the pathologic pathway.

### 4.2. Novel Therapeutic Approaches

Some of the new therapeutic strategies under evaluation involve the use of secretase modulators, immunotherapy, amyloid binders, metal-chelating agents, anti-inflammatory, and neuroprotective agents. Drugs targeting BACE1, like verubecestat, showed acceptable safety at doses that strongly reduce Aβ levels in plasma and CSF, but showed no cognitive or functional benefit [91]. γ-Secretase was another obvious target for inhibition with semagacestat. Unfortunately, target toxicity is inevitable due to the approximately 40 cellular substrates of the γ-secretase, leading to the closure of the clinical trials [92,93]. However, γ-secretase modulators [94,95] and γ-secretase stabilizers [96] are still being tested [10,16,83,86]. Immunotherapy, associated with specific passive immunization with monoclonal antibodies directed to Aβ peptides or Tau protein, has shown good results in the clearance of these proteins, as represented by the recently FDA-approved drug, aducanumab. Doig and coworkers referred to the reasons why small molecules and antibodies targeting Aβ oligomers have difficulties becoming effective AD therapies, resulting several times in failed attempts [97]. Nevertheless, other types of antibodies exist and have been studied as shown by Nguyen and colleagues [97,98]. Table 3 summarizes some of the current clinical trials [90,99,100] for AD therapeutics evaluation.

**Figure 7:**
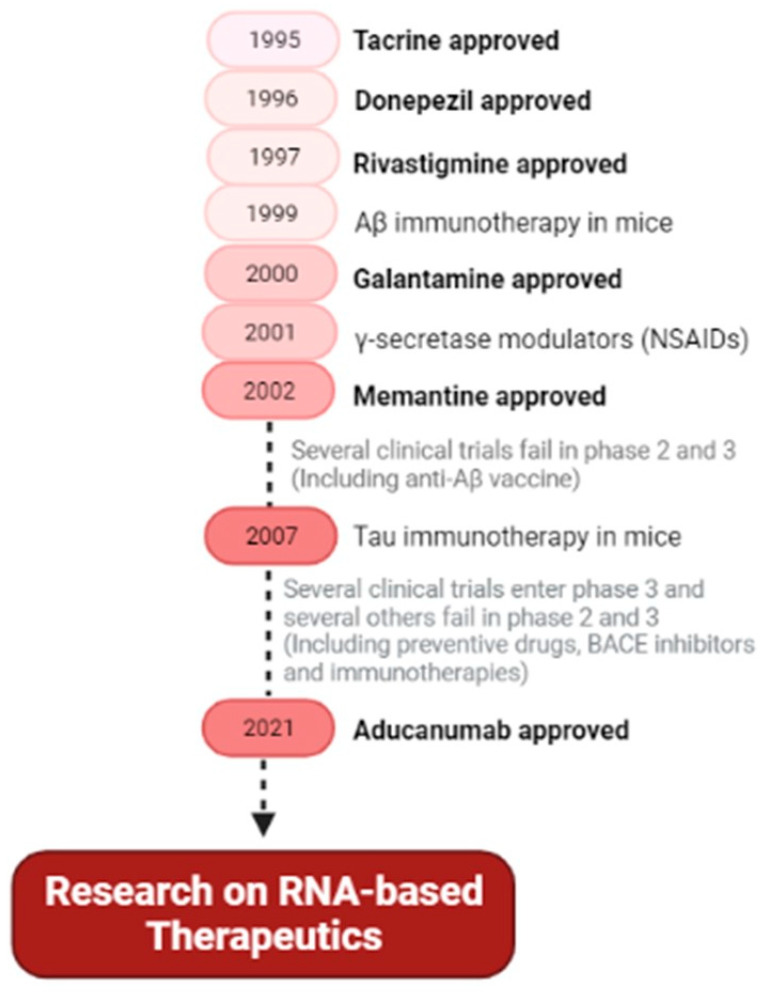
Main hallmarks and future perspective of therapeutic drugs for Alzheimer’s disease. Summary of the FDA-approved drugs for AD and the main types of drug research (secretase modulators, anti-Aβ vaccines, preventive drugs, and immunotherapies), and future therapeutic trends based on RNA. (NSAIDs—nonsteroidal anti-inflammatory drugs).

Therapeutic approaches under investigation for AD show to be effective in animal models but when reaching a clinical trial, the results are not successful. These failures are inevitable, but all research and information obtained so far can help in the identification of novel drug targets and the development of therapeutic strategies for this incurable disorder. Probably these outcomes are due to the complexity of AD, which is not overcome with single pathway approaches. So, maybe the next steps and future perspectives should be more focused on “multi-pathway” therapeutic strategies. Recent approaches point to RNA-based strategies, that offer great promise in the development of novel AD therapeutics. RNA-based strategies allow targeting a range of pathological features. Figure 7 shows the main events in AD therapeutic research, leading to the RNA research of today.

#### 4.2.1. RNA-Based Therapeutic Approaches

In recent years, RNA-based therapeutics have gained increased attention in the research field. This interest was mainly supported by the establishment of new RNA modifications that can improve the stability or on-target activity, and at the same time reduce off-target effects [101]. These characteristics transformed the former poorly performing RNA into the novel must-have therapeutic tools of tomorrow. In reality, it is expected that RNA therapeutics can overcome limitations associated with small-molecular inhibitors or antibiotics, enabling a higher target selectivity; regulation of gene expression and mRNA splicing; targeting ncRNAs that play important role in transcriptional and epigenetic regulation; and genome edition. Finally, it is important to mention that RNA-based therapeutics have been shown to have the rare ability to evolve pharmacologically with cancer mutation and pandemic viral infections [58,102]. As discussed for diagnosis, RNAs have also great potential as therapeutic agents and numerous studies are being developed, focusing on several types of RNAs, expecting the implementation of more effective treatments for uncurable diseases.

##### Coding RNAs

A new area that has recently been receiving huge attention is related to mRNA-based therapies. Compared to conventional gene delivery methods, these products showed some advantages which result in increased safety and efficiency, due to their characteristics. It should be highlighted that: mRNA will not be integrated into the host genome; mRNA does not need to enter the nucleus, being more effective in slowly- or non-dividing cells (neural cells); allows better control in protein expression, because promoter sequences or transcription is not necessary; and lastly mRNA does not contain sequences from viruses. Nevertheless, due to its linear structure, mRNA can be unstable under some physiological conditions and can be strongly immunogenic. To solve these problems, the development of a good delivery system is very important to reach therapeutic goals [103,104]. Lin and collaborators showed that mRNA can be used to express a non-secreted protein, Neprilysin, on the mouse brain. This membrane protein can degrade Aβ monomers and oligomers, resulting in a reduction in Aβ deposition. For the delivery of mRNA, the researchers have used self-assembled nano-micelles, that after releasing mRNA were degraded into nontoxic metabolites [103].

##### Small Non-Coding RNAs

siRNAs are synthetic double-stranded molecules that target complementary mRNA and can regulate gene expression through the assembly of the RNA-induced silencing complex (RISC). This technology is well studied and several chemical modifications are already known and available to increase siRNA stability and target selectivity. Several siRNA-based therapeutics were already approved by FDA for other diseases [58,105], supporting their potential use for AD therapeutics. McSwiggen and colleagues patented 325 siRNAs that target BACE, showing that some reduced BACE expression by 40–90% [106]. Kao and coworkers also designed siRNAs, where two of the siRNAs reduced BACE1 mRNA by more than 90% and Aβ production by 36–41%. Additionally, increasing neuroprotection against hydrogen peroxide-induced oxidative stress [107]. Different studies already described the decreased expression of APP, PSEN1, and PSEN2 after treatment with RNA interference, such as siRNA and shRNA [108]. As mentioned before lncRNA BACE1-AS is positively associated with BACE1 protein expression in vitro and in vivo, and knockdown of BACE1-AS by siRNA improved cognitive function in a mouse model of AD [72]. 

miRNA is one of the most characterized ncRNAs in AD. They can regulate mRNA translation by binding to the 3’untranslated region. This strategy allows the reduction in the amount of target protein, instead of only inhibiting their activity. miRNA can also target multiple genes, allowing targeting not only one pathologic pathway but the whole disease network [58,109,110]. As previously discussed, despite the requirements for tight control, this feature can also make a great difference in the complex and multifactorial AD. This characteristic makes the miRNAs an exciting new approach to AD therapeutics. Although this area is still in the early stages, studies have shown evidence of several miRNAs that target important molecules in this pathology [29,111,112,113,114]. Table 4 shows some studies of miRNAs in AD, that can have a potential application as AD therapeutic tools.

Besides miRNA, antimiRNA (inhibitors of endogenous miRNAs) and miRNA mimics are also molecules under evaluation for the development of AD therapeutics, exploiting their role in protein expression regulation. AntimiRNAs reduce complementary miRNAs levels to restore normal levels. As an example, microRNA-34c is increased in the hippocampus and blood of patients with AD, and its inhibitor enhances memory in AD mice models [138,139]. Lee and colleagues showed that AM206 inhibits miR-206 when injected into the cerebral ventricles of an AD mouse model, achieving an improved memory function as well as hippocampal neurogenesis and synaptic density [140]. On the other hand, miRNA mimics act like endogenous miRNA. For example, the Murphy group inhibited acetyl-CoA acyltransferase, in a mouse model of AD, using an artificial miRNA. This led to a reduction in Aβ plaques, cognition improvement, and reduced human APP levels [141]. MicroRNA-384 mimic downregulates the expression of APP and BACE1 in SH-SY5Y cells [142].

##### Long Non-Coding RNAs

In the CNS, lncRNAs are abundant and play a critical role in the pathogenesis of AD. This type of RNA can be highly specific for a sequence, cell, and tissue type, allowing for a specific regulation therapy. Their role is executed through epigenetic regulation of chromatin in cis, reversing or activating epigenetic modifications (mainly by DNA methylation). Since they are mainly in the nucleus besides functioning as scaffolds for chromatin modifiers, they can act also as transcriptional co-regulators. These roles allow lncRNAs to alter transcription, mRNA stability and influence the alternative splicing [58,109,143]. However, lncRNA-based therapeutic strategies are in an even earlier stage of understanding than miRNAs. So, in AD therapeutics, lncRNA are used as novel therapeutic targets for inhibition. Brain-derived neurotrophic factor antisense RNA (BDNF-AS) is a lncRNA that represses BDNF expression. The inhibition of BDNF-AS results in neuronal growth and differentiation [144]. Nuclear paraspeckle assembly transcript 1 (NEAT1), is involved in Aβ clearance by regulating the expression of endocytosis-related genes in AD. In an APP/PS1 transgenic mouse model, NEAT1 is increased and promotes the pathogenesis of AD via upregulating ubiquitination and degradation of PTEN-induced putative kinase 1 (PINK1), which provided a potential therapeutic strategy in AD [145,146]. As mentioned above, in Tg-19959 mice, knockdown of BACE1 or BACE1-AS transcripts causes reductions in BACE1 protein and insoluble Aβ [72]. Thus, therapies targeting the BACE1-AS transcript to reduced abundance of Aβ1-42 can already be envisioned. 

Also, circRNAs tend to be highly expressed in the brain. This accumulation is due to the normal aging process, commonly relating circRNAs to age-related diseases, such as AD [147]. Their role in AD remains unclear, but it is known that circRNAs can modulate the effect of miRNAs. Dysregulated circRNAs are associated with changed levels of downstream target mRNAs in mouse models, indicating that circRNA-microRNA-mRNA may play a significant role in the pathogenesis of AD [148]. Nevertheless, it has been shown that circRNAs can attenuate Aβ accumulation, neuroinflammation, oxidative stress and autophagy [42]. Additionally, studies have proven that circRNAs levels were altered in pre-symptomatic AD patients, which means that therapeutic interventions with these circRNAs may be a good option to treat preclinical AD [75]. Shi and coworkers showed that, in SH-SY5Y cells, ciRS-7 overexpression upregulated the ubiquitin carboxyl-terminal hydrolase L1 (UCHL1) protein, which accelerated APP and BACE1 degradation, reducing Aβ production [149]. In a different study, the Lu group reported that the circHDAC9 is significantly lower in the serum of AD patients. Naturally, circHDAC9 acts as a miR-138 sponge, reducing its levels and simultaneously increasing the expression of silent information regulator 1 (sirtuin1), which plays an important role in decreasing the accumulation of Aβ and attenuating mitochondrial dysfunction [150]. Thus, dysregulation in this circRNA could be related to the AD pathologic mechanisms. On other hand, overexpression of circNF1-419, in aged SAMP8 mice, enhances autophagy, reducing the levels of Tau, p-Tau, Aβ1-42 and APOE [151], also showing its relevance in AD.

##### Synthetic Oligonucleotides

A well-studied method of nucleic acids-based therapeutics is the use of antisense oligonucleotides (AOS). Short single-stranded synthetic oligonucleotides can control the expression of proteins in different ways. They can modulate the pre-miRNA splicing or bind to the mRNA, resulting in the repair of defective RNA or elimination of disease-associated proteins [101,152]. Ionis Pharma has patented AOS that target Tau expression and various regions of APP mRNA, inhibiting 39–82% of APP [101,153,154]. Banks and colleagues showed in SAMP8 mice that a radioactively tagged AO targeting the Aβ region of APP could transit the BBB and reversed the learning and memory deficits, possibly through reducing oxidative stress [155]. Chauhan and colleagues designed AOS that target the β-secretase cleavage site of APP and found that by administrating it into a mouse model of AD, the soluble APPα increased by 43%, and the soluble Aβ40 and Aβ42 levels decreased by 39% [156]. In another study, the Fiorini group administered AOS targeting PSEN1 to aged SAMP8 mice. The mice showed a reversal of learning and memory deficits and reduced brain oxidative stress biomarkers [157]. Caceres and colleagues showed that an AO targeting the 5’ end of the Tau gene, in the region before the start codon, can reduce the Tau protein level [158]. 

Aptamers are another class of molecules that have been exploited in the therapeutic perspective. Aptamers are short single-stranded oligonucleotides with a three-dimensional structure that bind to targets with high affinity and specificity [101]. Babu and colleagues developed an aptamer complexed with ruthenium that binds to Aβ oligomers inhibiting them [159]. On the other hand, the Liang group developed aptamers that bind to the extracellular domain of BACE1. When delivered in APP Swedish mutant cells, decreased Aβ40/Aβ42 levels and sAPPβ expression were found in comparison with untreated controls [160]. Kim and coworkers produced a Tau-1 aptamer that binds to Tau protein inhibiting its oligomerization, thus reducing the levels of oligomeric Tau by approximately 94% [161].

In general, numerous studies have focused on finding potential treatments using RNAs and even though, the transition from the laboratory bench to clinical trials still is a challenge. 

#### 4.2.2. Challenges in the RNA-Based Therapeutic Applications

The ongoing research on RNA-based technology demonstrates that RNA possesses attractive characteristics to be used in therapy. The ability of RNA to induce a robust silencing of targeted genes expression and the possibility to promote long-lasting therapies is already recognized. Some recent studies also suggest that the dosage required for RNA therapeutics can be low, which can reduce the occurrence of undesirable adverse effects in the patients, one of the biggest problems encountered in the development of therapeutics [162,163]. Therapeutic oligonucleotides composed of naturally occurring nucleotides are rapidly degraded in vivo or suffer renal clearance, which makes them unsuitable for drug development. For pharmacological applications, certain characteristics must be considered, like product stability, safety, and biological activity [58,101]. So, RNA-based technologies typically use synthetic oligonucleotides around 8–50 nucleotides in length, produced by chemical synthesis. The success rate of RNA synthesis depends on the sequential deprotection-coupling and oxidation reaction followed by purification, usually performed by high-performance liquid chromatography (HPLC). In this production method, the addition of novel chemical modifications is possible and one can exploit conjugation strategies to improve RNA pharmacokinetics and tissue-specific delivery. 2’-O-methyl (2’-OMe) RNA, 2’-fluoro (2’ F) RNA, 2’-O-methoxyethyl (2’-MOE) RNA, PEGylated drugs are some of the modifications successfully incorporated in FDA-approved oligonucleotide drugs. These modifications can improve RNA stability and bioavailability (resistance to nucleolytic degradation or renal clearance) [101]. Another strategy is the synthesis of neutral siRNA, masking the negative charge on the phosphate backbone to reduced renal clearance [164]. However, there is a risk that these chemical modifications may alert the cells to see RNA as an exogenous or pathogenic element or even amplify off-target effects due to the changed structure, which can lead to changes in functional properties. Another limitation is that the process of synthesizing longer sequences is more prone to errors and can lead to changes in functional properties or toxicity to the cell. The increased cost of synthetic RNAs depending on their length and number of modifications, and the fact that the synthetic RNA is provided on a micromolar scale may influence the decision to use RNA in clinical investigations [165,166].

Other solutions to this production method are enzymatic synthesis and recombinant biosynthesis. The first is a well-established method, based on the use of bacteriophage systems to produce RNA molecules from DNA sequences by in vitro transcription. Preparative polyacrylamide gel electrophoresis and anion exchange fast protein liquid chromatography are then frequently used to further purify the RNA products. Some of the disadvantages of enzymatic production are the heterogeneity at 3’ and 5’ ends of the products, the decreased reliability of RNA polymerase as the transcript length increases and the absence of post-transcriptional modification machinery. The greater advantage of this method is the vast commercialized kits available, the versatility to generate RNA molecules of various lengths in the microgram to milligram amounts [165,167,168]. Lastly, recombinant production is achieved by using host cells modified with an effective plasmid DNA (pDNA) coding for the target RNA sequences. The cells growth occurs in a culture medium replicating the pDNA molecule and expressing the target RNA sequences. The main challenges of these methods are the difficulty in purifying the samples and the fact that the RNA is highly susceptible to RNases activity in the culture medium. Nevertheless, this is the most cost-effective approach [165,166,169,170]. Currently, only chemical and enzymatic syntheses are approved by Food and Drug Administration (FDA). 

Besides production and purification, another big challenge for RNA application as therapeutics is the stable delivery of RNAs and the entrance into the cells. To accomplish this, several options have been tried to encapsulate, protect, and deliver RNAs. Two main strategies have been exploited as delivery methods, including the non-viral methods, based on the use of lipid-based or polymeric nanoparticles, and viral vectors such as adenovirus or adeno-associated viruses. The viral vectors are more powerful at transfection but present immunogenicity, potential toxicity, the possibility of activating oncogenes and difficulty in increased production. On the other hand, the non-viral vectors have an easy and reproducible method of production, present higher biocompatibility, biodegradability, non-toxicity, and non-immunogenicity. Giving these characteristics, they are preferred over the viral vectors [171,172,173]. 

More specifically, in the treatment of brain disorders, the main obstacle of applying RNA therapeutics is the existence of the BBB, because therapeutic RNAs are typically too large to cross the BBB. The BBB is a specialized structural, physiological, and biochemical barrier made of a highly specialized endothelial cell membrane that lines the brain microvasculature and regulates the movement of molecules from the blood to the brain, maintaining and protecting the ideal neuronal functioning from neurotoxins [39,41,174,175]. This blood–brain interface also allows exporting of potentially neurotoxic molecules from the brain to the blood, such as Aβ peptides [37,39,174]. 

As referred above, the BBB is the main barrier that prevents therapeutic molecules from entering the brain [37]. Even with the disruption of the BBB, therapeutic agents such as anti-amyloid monoclonal antibodies continue to have limited brain penetration; it is estimated that less than 1.5% of an administered dose enters the brain [37,40]. Due to these obstacles, progresses in the development of new drugs for AD has been slow. Despite this, there are some FDA-approved treatments for the brain region, such as Bevacizumab (Avastin) and Natalizumab (Tysabri) (monoclonal antibodies for brain, cancer and multiple sclerosis), that are not able to cross the BBB with an effective therapeutic concentration [176]. The first and the only class of small molecules approved for the treatment of AD that managed to cross the BBB were acetylcholinesterase inhibitors, with a molecular weight between 198 and 380 Da [177]. Some studies for the treatment of AD have focused on inhibitors of secretase that give rise to the Aβ peptide. However, due to their size and chemical properties their access to the brain was very limited [177]. Nevertheless, in 2019, a hope emerged when adeno-associated virus-9-based gene therapy, a one-time intravenous administration of the self-complementary-AAV9 encoding the survival motor neuron type 1 gene, was approved for the treatment of children spinal muscle atrophy-1. This is the first FDA-approved biotech product for a brain disease that crosses the BBB [178,179]. This result gives hope for brain therapies, such as AD, and shows that it is possible to overcome this complex and great obstacle. However, as discussed above the challenges imposed by viral vectors emphasize the need to develop non-viral vectors that can efficiently deliver genes to the brain through systemic injection [180]. Gene therapy has increasingly shown great potential for the treatment of AD, however, due to the presence of BBB, non-viral vectors are less effective to be delivered into the brain through systemic administration [180,181]. In 2019, inspiring results emerged to surpass the BBB. Guo and coworkers developed the gene carriers composed of cationic polymers, PEGylated poly(2-(*N*,*N* dimethylamino) ethyl methacrylate) (PEG-PDMAEMA), surface-modified with both BBB targeting ligand (CGN peptide) and Aβ-targeting ligand (QSH peptide) and verified that these complexes penetrated the barrier and specifically delivered siRNA to neurons close to the amyloid plaques. They observed that these complexes not only reduced the Aβ plaques, but also slowed down the neurodegeneration process, thus promoting the cognitive performance of AD [180]. In addition, in 2020, Zhou and colleagues developed a glycosylated nano-delivery system, which uses glycemia-controlled glucose transporter-1 recycling to facilitate the nanomedicine BBB penetration, for more effective AD therapy. This system combined with a siRNA led to decreased expression of BACE1, consequently leading to reduced levels of Aβ plaques, with the added benefit of suppressed levels of phosphorylated Tau protein. In addition to the excellent biocompatibility, blood stability and effective BBB penetration, these nanoparticles also exerted high brain accumulation [181]. 

Another strategy that is being exploited to overcome the BBB passage problem is the intracerebral injection of drugs. In this strategy, the drug can be injected into the brain through a burr hole drilled in the skull. However, besides the invasiveness, drug delivery is mainly confined to the injection site, due to limited diffusion within the brain [176]. Another of the solutions and one of the most currently explored is nasal delivery to the brain. Small fat-soluble molecules enter the cerebrospinal fluid olfactory after nasal administration, diffusing first through the nasal epithelial barrier, later through the olfactory arachnoid membrane, and finally the brain. However, despite 40 years of studies in the delivery of transnasal drugs to the brain, there is still no biological drug that has been approved by the FDA for the treatment of AD after intranasal delivery. The main obstacle to overcome in this method is the large volumes of the therapeutic agent needed, which causes local damage to the nasal membranes [176]. However, once this problem has been surpassed, it can be a very promising approach for the treatment of AD, being painless, and enabling a prolonged treatment without huge costs and difficulties. 

**Figure 8:**
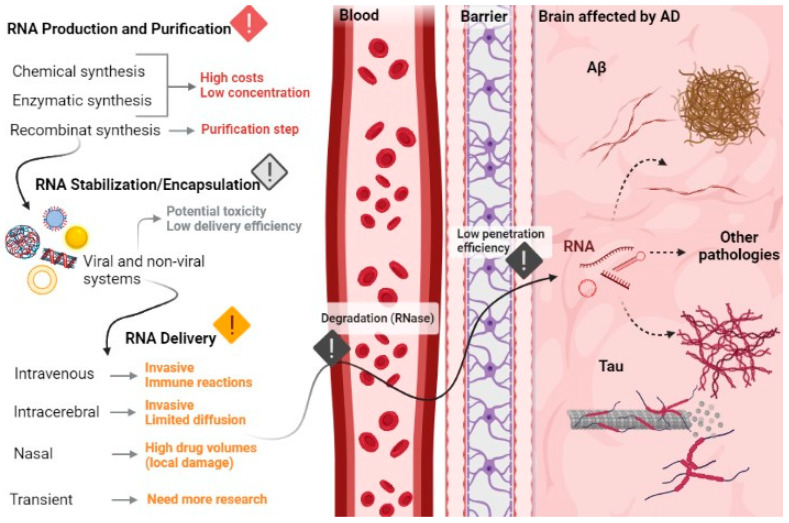
Overview of the main challenges in the development of RNA-based therapeutics, from production until brain cells delivery.

In addition, a different strategy under study that has already shown some interesting results is the transient delivery of drugs to the brain by ultrasonic irradiation of the brain after intravenous administration of microbubbles [176,182]. This noninvasive method causes a transient rupture of the BBB and provides an exciting opportunity for focused ultrasound (FUS) coupling research in targeted drug delivery, immunotherapy, stem cell gene therapy into various complex and deep brain structures, including the hippocampus [176,182]. An example of the use of this strategy is the study carried out in 2020 by Rezai and co-workers, where they used magnetic resonance (MR)-guided low-intensity focused ultrasound (FUS) to surpass BBB. The research group found that FUS technology and focal BBB opening offer a unique opportunity for targeted delivery of therapeutics to meaningful volumes of essential brain structures in AD and other neurological conditions [182]. More results on focused ultrasound applications are described in Nguyen and team review [98]. Thus, underestimating drug delivery through the BBB and just looking for biomolecules for the treatment of AD is not the way to successfully find an effective treatment for AD. The development of new biological treatments for AD and other brain diseases, which may eventually be approved by the FDA, will require an effort and an integrated approach to innovation in terms of drugs and delivery strategies [176]. In addition, intracellular barriers such as unspecific RNA delivery, inefficient cellular uptake and intracellular processing of target RNAs in endosomes need to be overcome. Extracellular barriers are also a major obstacle to RNA delivery and application, as they are responsible for the low bioavailability of circulating RNA, for the enzymatic degradation by nucleases in the bloodstream, for rapid renal release, for phagocytosis, opsonization by blood, diffusion through the cell matrix and, finally, undesirable toxicity due to an immune response and/or unwanted effects [172]. Figure 8 summarizes the principal challenges in the application of RNA therapeutics in AD. 

In general, RNA-based therapies have been evolving in the last few years, both in AD and other diseases. However, there are still obstacles that must be overcome to achieve an effective and suitable therapy for human use. Thus, the production, purification, stabilization, and delivery of these biopharmaceuticals are the main points that need to be addressed and enhanced to reach that goal.

## 5. Conclusions and Trends in AD Diagnosis and Treatment

AD is one of the neurodegenerative diseases that most affect people in the world, having just a few therapies to relieve symptoms, and all of them with adverse side effects. Presently, only one novel treatment managed to gain FDA approval to address the pathological features. Despite the efforts, and although many drugs have shown success in cell and animal models, the results often cannot be replicated in human trials. These limitations are due to the complex and interconnected pathological mechanisms that can directly or indirectly result in the hallmarks of AD. Thus, an alternative path is the accessible and non-invasive early diagnosis of AD. If therapies take a long time to achieve therapeutic standards, a successful pre-clinical diagnosis of AD can perhaps be a helpful manner to change the outcome of the AD. Additionally, searching for novel biomarkers can open the path to the identification of new target molecules, which can lead to different approaches for AD treatment. In conclusion, there is a continuous and unmet need for better diagnostic and therapeutic strategies, and RNA-based tools offer major advantages over traditional ones. Looking at the big picture, the recent discoveries and studies show not only that we are at the threshold of a new RNA-based diagnostic and therapeutic era, but also that this area has the potential to dominate the future of biomedical and clinical applications.

## Figures and Tables

**Table 1:** Suggested circulating miRNA profiles specific for AD, detected in blood (Adapted from Leidinger and collaborators [63]).

miRNA	Expression in AD
brain-miR-112 (Unknown)	Upregulated
brain-miR-161 (Unknown)	Upregulated
hsa-let-7d-3p	Upregulated
hsa-miR-5010-3p	Upregulated
hsa-miR-26a-5p	Upregulated
hsa-miR-1285-5p	Upregulated
hsa-miR-151a-3p	Upregulated
hsa-miR-103a-3p	Downregulated
hsa-miR-107	Downregulated
hsa-miR-532-5p	Downregulated
hsa-miR-26b-5p	Downregulated
hsa-let-7f-5p	Downregulated

**Table 2:** Clinical trials showing the potential diagnostic of AD using miRNAs as biomarkers.

Institutions	miRNA	Sample Type	Disease	Status
Shanghai Mental Health Center	miRNA 107	Plasma	MCI ^1^	Unknown
		CSF ^1^	AD ^1^	
Sun Yat-sen University	miRNAs	Blood	MCI ^1^	Unknown
			AD ^1^	
Seoul National University Hospital	miRNA 206	Olfactory neuroepithelium tissue	AD ^1^	Completed
Shanghai Mental Health Center	miRNAs	Plasma	MCI ^1^ due to AD ^1^	Recruiting
			AD ^1^	
Shanghai Mental Health Center	miRNAs	Plasma	MCI ^1^ due to AD ^1^	Not yet recruiting
			Mild AD ^1^	
			Moderate AD ^1^	
			Severe AD ^1^	
			LBD ^1^	
			FTD ^1^	
Neuromed IRCCS	miRNAs	Blood	MS ^1^	Unknown
		CSF^1^	PD ^1^	
			ALS ^1^	
			AD ^1^	
University of Pisa	miRNA-30	Blood	PD ^1^	Completed
	miRNA-7		AD ^1^	

^1^ CSF—Cerebrospinal fluid; MCI—Mild cognitive impairment; AD—Alzheimer’s disease; LBD—Lewy body dementia; FTD—Frontotemporal dementia; MS—Multiple sclerosis; ALS—Amyotrophic lateral sclerosis; PD—Parkinson’s Disease.

**Table 3:** Potential therapeutic drugs and applications in AD, currently in clinical trials.

Drug	Definition	Expected Results	Phase
BAN2401	Human monoclonal antibody Affinity for soluble Aβ ^1^ protofibrils	Human monoclonal antibody Reduction in Aβ ^1^ levels and cognitive decline	Phase 3
Gantenerumab	Human monoclonal IgG1 antibody	Reduction in the Aβ ^1^ plaques	Phase 3
	Affinity for Aβ ^1^ aggregated forms		
TRx0237	Second generation Tau aggregation inhibitor	Prevention of Tau aggregation	Phase 3
		Dissolution of existing Tau aggregates	
ALZT-OP1	Cromolyn and ibuprofen (anti-inflammatory compounds)	Reduction in neuroinflammation	Phase 3
		Clearance of Aβ ^1^	
COR388	Gingipains inhibitor (Virulence proteases from *Porphyromonas gingivalis*, common in AD ^1^ brains)	Reduction in Aβ ^1^ 42 production, neuroinflammation, and hippocampal degeneration	Phase 2/3
Masitinib	Selective tyrosine kinase inhibitor	Modulation of neuroinflammation	Phase 3
AGB101 (Levetiracetam)	SV2A ^1^ modulator (anti-convulsant medication)	Reduction in Aβ ^1^-induced cognitive and functional impairment	Phase 3
Blarcamesine	Sigma-1 chaperone receptor agonist	Prevention of memory loss	Phase 2/3
		Neuroprotective effects	
		Blockage of Tau hyperphosphorylation	
Troriluzole	Prodrug conjugate of riluzole (mechanism of action is not fully understood)	Inhibition of glutamate release	Phase 2/3

^1^ Aβ—β-amyloid peptide; AD—Alzheimer’s disease; SV2A—Synaptic vesicle glycoprotein 2A.

**Table 4:** Studies showing the therapeutic potential of miRNA in AD.

miRNA	Target Proteins	Therapeutic Potential	References
miRNA-9-5p	GSK-3β ^1^	Inhibition of mitochondrial damage and oxidative stress	[115]
miRNA-15b	NF-κB ^1^ signaling	Inhibition of BACE1 ^1^, APP ^1^ and Aβ ^1^ levels	[116]
	BACE1 ^1^		
miRNA-21	PDCD4 ^1^/ PI3K ^1^/AKT ^1^/GSK-3β ^1^ pathway	Inhibition of Aβ ^1^-apoptosis induced	[117]
miRNA-29a/b-1	BACE1 ^1^	Regulation of BACE1 ^1^ and Aβ ^1^ levels	[118]
miRNA-29c	BACE1 ^1^	Reduction in BACE1 ^1^ and Aβ ^1^ levels	[119,120]
	PKA ^1^/CREB ^1^	Neuroprotection	
miRNA-34a-5p	BACE1 ^1^	Inhibition of Aβ ^1^-induced apoptosis and oxidative stress	[121]
miRNA-31	APP ^1^	Improvement of cognition and memory deficits	[122]
	BACE1 ^1^	Reduction in glutamate vesicles accumulation	
		Reduction in APP ^1^, BACE1 ^1^ and Aβ ^1^	
miRNA-101a-3p	APP ^1^	Regulation in APP ^1^ and Aβ ^1^ levels	[123]
miRNA-98	HEY2 ^1^	Inactivation of Notch signaling pathway	[124]
miRNA-101	APP ^1^	Reduction in APP ^1^ and Aβ ^1^ levels	[125]
miRNA-106b	Fyn ^1^	Inhibition of Aβ ^1^1-42-induced Tau phosphorylation at Tyr18 ^1^	[126]
miRNA-107	BACE1 ^1^	Inhibition of BACE1 ^1^	[127]
miRNA-124-3p	CAV1-PI3K/Akt/GSK3β ^1^ pathway	Attenuation of cell and abnormal Tau hyperphosphorylation	[128]
miRNA-125b-5p	BACE1 ^1^	Inhibition of Aβ ^1^-induced apoptosis and oxidative stress	[121]
miRNA-137	SPT ^1^	Inhibition of Aβ ^1^ levels	[129]
miRNA-153	APP ^1^	Reduction in APP ^1^ levels	[130]
miRNA-181c	SPT ^1^	Inhibition of Aβ ^1^ levels	[129]
miRNA-195	BACE1 ^1^	Inhibition of BACE1 ^1^ and Aβ ^1^ levels	[131]
miRNA-200a-3p	Bax ^1^/CASP3 ^1^ axis	Inhibition of apoptosis, Aβ ^1^ and p-Tau levels	[132]
	BACE1 ^1^		
	PKA ^1^		
miRNA-200b/c	PS6KB1 ^1^ (Insulin signaling)	Reduction in Aβ ^1^ secretion relieved and memory impairments	[133]
miRNA-298	BACE1 ^1^	Repression of APP ^1^, BACE1 ^1^, Aβ ^1^ and some Tau forms	[134]
	APP ^1^		
	Tau under study		
miRNA-326	VAV1 ^1^	Inhibition of Aβ ^1^ deposition, apoptosis, Tau phosphorylation	[135]
miRNA-328	BACE1 ^1^	Regulation of BACE1 ^1^ expression	[136]
miRNA-339-5p	BACE1 ^1^	Inhibition of BACE1 ^1^ expression	[137]

^1^ GSK-3β—glycogen synthase kinase 3 beta; NF-κB—nuclear factor kappa B; PDCD4—programmed Cell Death 4 gene; PI3K—phosphoinositide 3-kinase; AKT—protein kinase B; BACE1—β-secretase; APP—amyloid precursor protein; Aβ—β-amyloid peptide; PKA—protein kinase A; CREB—cAMP response element-binding protein; HEY2—Hes related family BHLH transcription factor with YRPW motif 2; Tyr18—tyrosine residues 18; CAV1—caveolin-1; SPT—serine palmitoyltransferase; Bax—BCL2 associated X, apoptosis regulator; CASP3—caspase-3; p-Tau—phosphor-Tau; PS6KB1—protein S6 kinase B1; VAV1—Vav guanine nucleotide exchange factor 1.
